# Global research status and trends of bronchiectasis in children from 2003 to 2022: A 20-year bibliometric analysis

**DOI:** 10.3389/fped.2023.1095452

**Published:** 2023-02-03

**Authors:** Ting Gong, Xueer Wang, Shuanglian Li, Li Zhong, Lin Zhu, Tingting Luo, Daiyin Tian

**Affiliations:** Department of Respiratory Disease, Children’s Hospital of Chongqing Medical University, Chongqing Key Laboratory of Pediatrics, Ministry of Education Key Laboratory of Child Development and Disorders, National Clinical Research Center for Child Health and Disorders, China International Science and Technology Cooperation Base of Child Development and Critical Disorders, Chongqing, China

**Keywords:** bronchiectasis, children, pediatrics, bibliometric, hotspot, CiteSpace, VOSviewer

## Abstract

**Background:**

This study aims to analyze the research hotspots, evolution, and developing trends in pediatric bronchiectasis over the past 20 years using bibliometric analysis and visualization tools to identify potential new research directions.

**Methods:**

Publications related to bronchiectasis in children were retrieved from the Web of Science Core Collection (WoSCC) database from 2003 to 2022. Knowledge maps were performed through VOSviewer1.6.18 and CiteSpace6.1 R2.

**Results:**

A total of 2,133 publications were searched, while only 1,351 original articles written in English between 2003 and 2022 were incorporated. After removing duplicates, we finally included 1,350 articles published by 6,593 authors from 1,865 institutions in 80 countries/regions in 384 different academic journals with an average citation frequency of 24.91 times. The number of publications shows an extremely obvious binomial growth trend. The majority of publications originated from the United States, Australia, and England. The institutes in Australia, especially Charles Darwin University, published the most articles associated with pediatric bronchiectasis. In addition, *Pediatric Pulmonology* was the most published journal. In terms of authors, Chang AB was the most productive author, while Gangell CL had the highest average citation frequency. The five keywords that have appeared most frequently during the last two decades were “children,” “cystic fibrosis,” “bronchiectasis,” “ct,” and “pulmonary-function.” According to keyword analysis, early diagnosis and intervention and optimal long-term pediatric-specific management were the most concerned topics for researchers.

**Conclusion:**

This bibliometric analysis indicates that bronchiectasis in children has drawn increasing attention in the last two decades as its recognition continues to rise, providing scholars in the field with significant information on current topical issues and research frontiers.

## Introduction

Bronchiectasis is a chronic bronchial disease caused by damage to the muscle and elastic tissues of the proximal bronchial wall, resulting in deformation and irreversible dilatation of the bronchial wall, which can lead to chronic respiratory insufficiency and respiratory morbidity and mortality. The pathological changes in pediatric bronchiectasis manifest in a vicious cycle of impaired airway clearance, airway inflammation, and repeated lung deterioration, leading to progressive and irreversible damage to the lung structures (1, 2). Chronic wet or productive cough with recurrent pulmonary exacerbations is the dominant symptom of bronchiectasis in children due to poor expectoration. The diagnosis of bronchiectasis is confirmed by chest high-resolution computed tomography (cHRCT) scanning with the bronchoarterial ratio ≥0.8 in children (3, 4).

Bronchiectasis is experiencing a clinical and research renaissance, which is considered an emerging global epidemic (5). With the increasing awareness of the disease and advancements in imaging technology, the overall incidence in children is rising, ranging from 0.2 to 753 per 100,000 (6). The incidence varies greatly in different regions and populations, which is higher in undeveloped countries compared to high-income countries (7). However, the incidence of indigenous children in Australia, New Zealand, Canada, and Alaska is much higher than in other groups (8–11).

There are numerous underlying causes of bronchiectasis. However, in up to half of all cases, the cause cannot be identified (idiopathic). The known causes can be divided into genetic and acquired. Major genetic diseases associated with bronchiectasis include cystic fibrosis (CF), primary ciliary dyskinesia (PCD), primary immunodeficiencies, or other rare disorders. Major acquired causes are severe bacterial infections or postinfectious bronchiolitis obliterans (12). As we know, bronchiectasis can be divided into two categories according to the etiology: noncystic fibrosis bronchiectasis (NCFB) and cystic fibrosis bronchiectasis (CFB). NCFB is considered an orphan disease with increasing prevalence in recent years, which is thought to be related to the high disease burden of children and has brought huge economic costs (13).

Bronchiectasis is the end consequence of many conditions related to recurrent or persistent respiratory infections or both. In the adult population, idiopathy was the most common cause, followed by postinfectious. Other common causes include chronic obstructive pulmonary disease (COPD), asthma, immunodeficiency, and rheumatic diseases (14). Unlike in adults, the most common cause of NCFB was postinfectious, followed by PCD and immunodeficiency in children (15). In addition, aspiration/foreign body and congenital malformation of respiratory tracts are also potential causes (16).

Because of the lack of attention to pediatric bronchiectasis compared with other chronic respiratory diseases (17), studies in children on epidemiology, associated etiology, clinical diagnosis and management, or negative impacts on growth are still too scarce to support advanced research (18). Therefore, the current treatment approaches in children are largely based on adult and CF studies (19). Although bronchiectasis in children is partially similar to adults in terms of etiology and symptoms, it differs in comorbidity, potential etiology, age-related immunoreaction, pathogen pattern changes, and therapeutic outcomes (20–23). Bronchiectasis was once considered irreversible and progressive, but studies have found that the disease progression in mild pediatric bronchiectasis can be halted and even reversed with optimal clinical management (24, 25). Therefore, adult bronchiectasis data may not be fully applicable to children, and in such an era of big data in medical research, it is essential to form a pediatric-specific standard. Bibliometric analysis may be a solution.

Bibliometrics is a statistical tool to quantitatively analyze published articles based on research methods such as mathematics and statistics, which enables scholars to intuitively grasp the main priorities to guide their advanced research (26–29). CiteSpace supports the visual exploration of current scientific knowledge (30), and VOSviewer offers bibliometric maps based on collaborative or co-occurrence data for a comprehensive outlook of the whole structure (31, 32). For large volumes of information to be analyzed to depict dynamic development in this field, a bibliometric analysis is of critical necessity. However, no bibliometric analysis of publications on pediatric bronchiectasis has been published until now.

Therefore, our study is performed on time, aiming to assess the current status of pediatric bronchiectasis and provide ideas for potential research directions. In addition to evaluating research trends by visualizing the contributions of countries, institutions, journals, and authors, our bibliometric analysis also concentrates on cooperation and co-occurrence relationships, achieving an in-depth exploration of research hotspots and a precise prediction of future development in this field.

## Methods

### Data collection

The Web of Science Core Collection (WoSCC) database is considered to be the paramount data source for bibliometric analysis because of its more consistent and standardized records compared to other databases (33). In addition to having built-in analysis capabilities, WOS also allows for exporting its data to other programs for additional analysis. That is why we performed a systematic search of the literature within the Web of Science database using the following keywords: bronchiectasis, children, pediatric, infant, and adolescent. The search was carried out by using the combination of the above keywords and their free words. The advanced search strategies were conducted as follows: [TS = (bronchiectas* OR bronchodilation OR bronchial dila*)] AND [TS = (child* OR pediatric* OR paediatric* OR infant* OR adolescent* OR neonate* OR toddler*)]. Two authors (TG and XW) independently searched the WoSCC database for relevant literature, set the limitation of the English language and the article type with a time span from 1 January 2003 to 12 December 2022, and screened for duplicates. TG and XW assessed the credibility of these potential articles by evaluating the title and abstract of articles to determine whether the literature should be included or excluded. We downloaded the full text, conducted a more detailed evaluation of the uncertain literature, and resolved the differences after consulting and discussing with the senior author (DYT). Finally, 1,351 articles were downloaded for records and cited references in the document format of plain text files. Given that only the “download_*.txt” format could be recognized by CiteSpace, the files were renamed before being imported into CiteSpace 6.1.R2 for advanced analysis. At last, useful data were extracted independently and reviewed by the senior author. Considering that frequent database updates may cause bias, all literature retrieval and data collection were conducted within 1 day on 12 December 2022. What is more, all data in this study were directly exported from public databases, so ethical approval was not needed. The detailed retrieval procedure is presented in Table 1.

**Table 1:** Data sources and flow of the retrieval strategy.

Content			
Data sources	Web of Science Core Collection
Publication date	1 January 2003–12 December 2022
Languages	English only
Document type	Article only
Search strategy			
	#1	11,013	TS = (bronchiectas* OR bronchodilation OR bronchial dila*)
	#2	1,720,817	TS = (child* OR pediatric* OR paediatric* OR infant* OR adolescent* OR neonate* OR toddler*)
	#3	2,133	#1 AND #2
	#4		Document types: (Article)
	#5		Language: (English)
	#6		Publication year: (2003–2022)
	#7	1,351	#1 AND #2 AND #3 AND #4 AND #5 AND #6

### Statistical analysis

Considering that different bibliometric software have distinguished characteristics (34), we combined VOSviewer1.6.18, CiteSpace 6.1.R2, Microsoft Excel 2021, and R 4.2.1 to meet our actual requirements. Full records and cited references of all articles were obtained from WoSCC, which included all the bibliometric fields that we can extract to analyze, such as title, author, keywords, publication year, country, journal, institution, citations, references, and so on.

Co-authorship, co-occurrence, citation, bibliographic coupling, co-citation analysis among authors, countries, institutions, and the co-citation network maps of journals were constructed by VOSviewer. In the generated network maps, node size represents the number of articles or citations and the width of a link between nodes represents the strength of cooperation or co-citation. Moreover, the total link strength is related to the cooperation intensity. VOSviewer was utilized to build visual network maps that simplified the complex data into concise figures to realize an intuitive and comprehensive understanding of the structure.

Likewise, CiteSpace 6.1.R2 was used to not only perform co-occurrence analysis of countries, institutions, journals, and keywords after pruning with time slicing of 1 year per slice from 2003 to 2022 but also generate the keyword timeline map and dual-map overlay. In addition, the burst dictation and node centrality calculation were conducted by CiteSpace 6.1.R2. After clustering by keywords, co-occurrence networks will be divided into different clusters denoting different research themes and labeled by the terms extracted from relevant data. In the co-occurrence maps, node size indicates the co-occurrence intensity or the citation frequency, and the link indicates a co-occurrence or cooperation relationship between two nodes. A node with a red outer ring denotes a citation burst, which means the citations increased sharply within a short period of time, while a purple outer ring represents the degree of betweenness centrality, which means the linking strength of nodes or clusters. It is worth noting that burst nodes are associated with merging research hotspots and nodes with high betweenness centrality have close connections with other nodes. They require high priority because of their vital importance in bibliometric analysis, which may indicate the future trends in basic and clinical research in this field (35).

Microsoft Excel 2021 was used to manage the corresponding data and generate bar charts and line charts of the number of publications. R4.1.2 was used to produce the annual publication and citation count of authors, showing the changes in production over time.

## Results

### Annual publication outputs and trends

**Figure 1:**
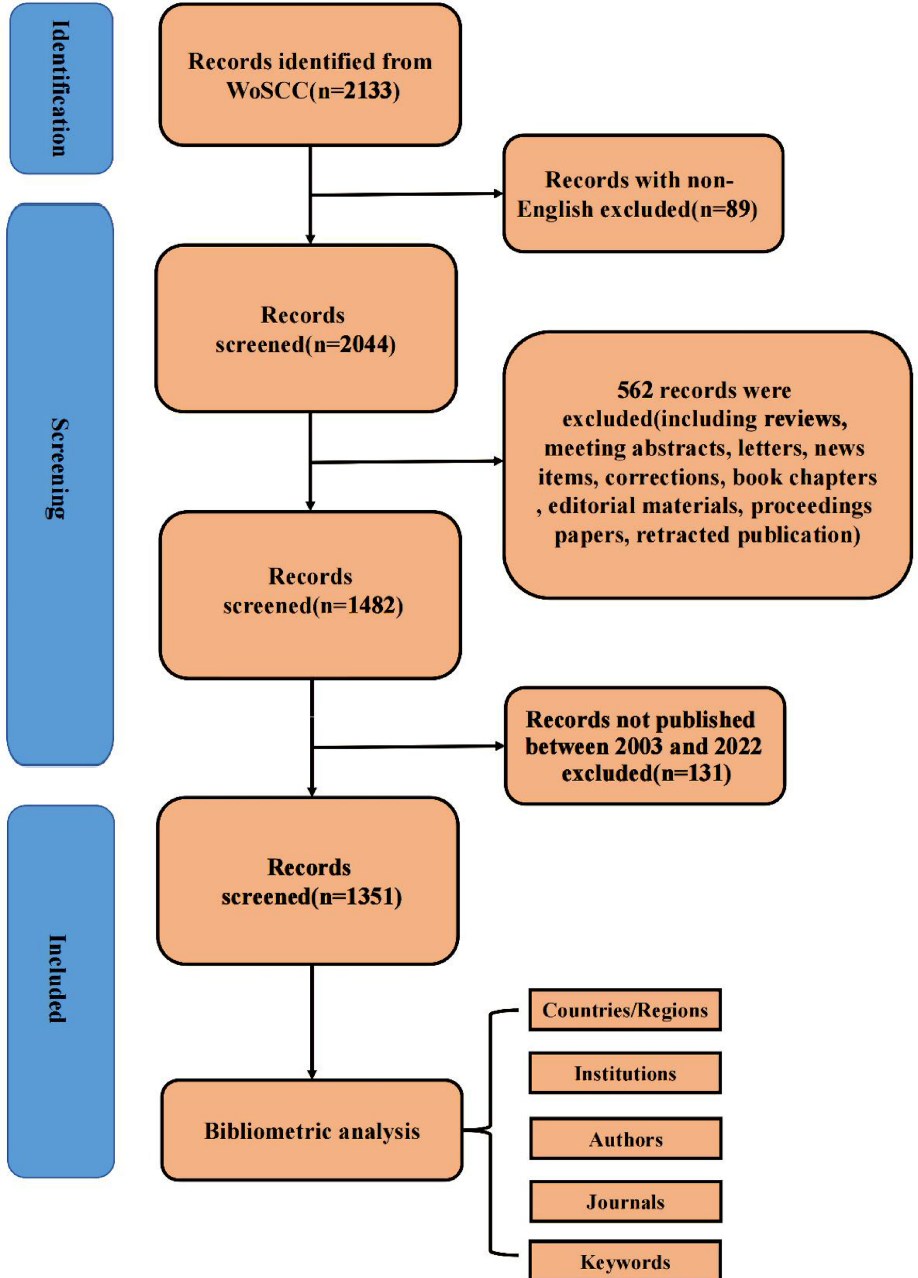
Flowchart of literature screening.

**Figure 2:**
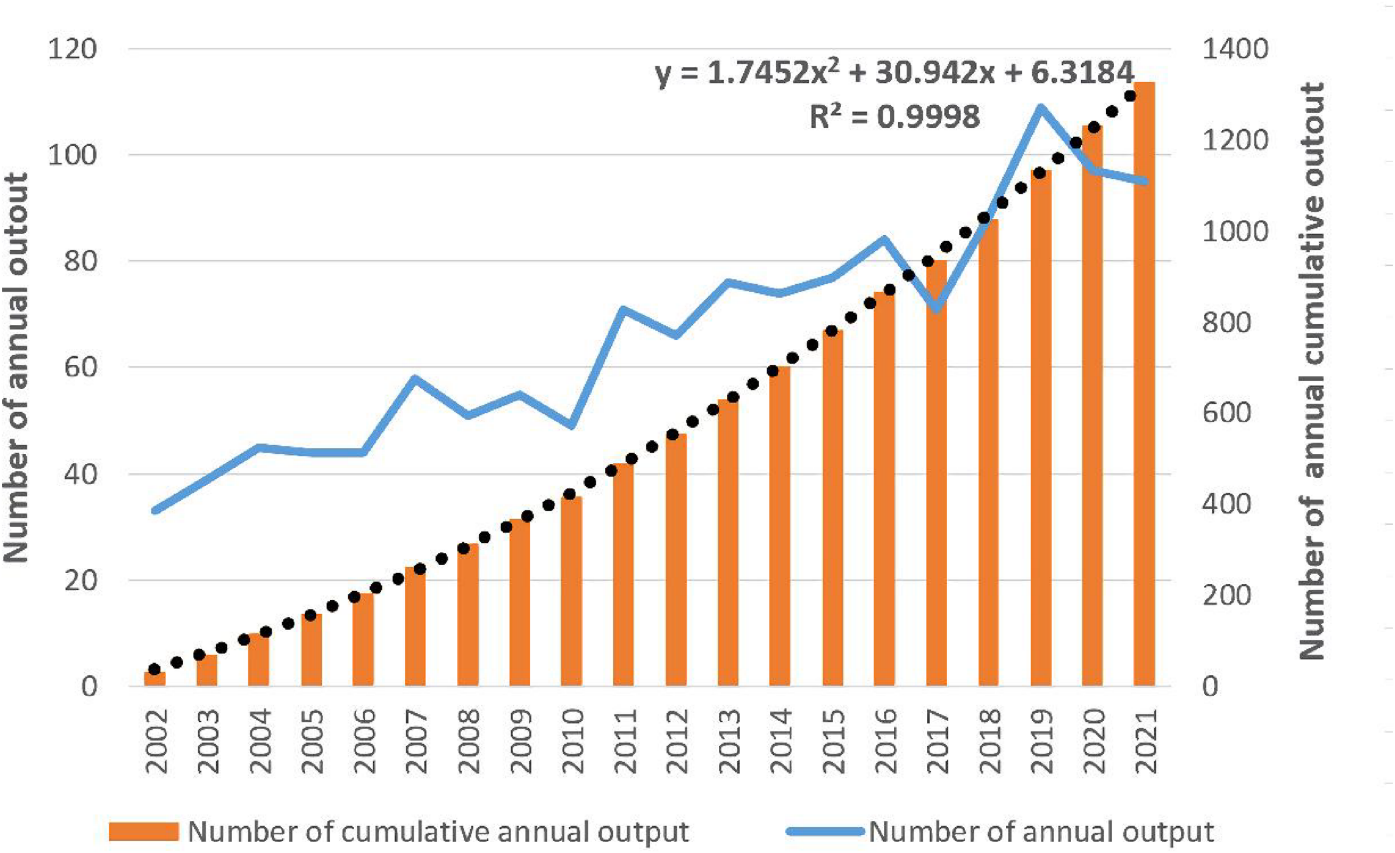
Trend chart of the number of publications on pediatric bronchiectasis between 2003 and 2022.

As shown in Figure 1, we retrieved 2,133 publications related to bronchiectasis in children from 2003 to 2022. After setting the limitations of the English language and article type and removing the duplicates, only 1,351 articles were involved in analysis. The publication number of relevant articles increased from 34 in 2003 to 95 in 2022. A minor decline in annual publications was seen in 2011 and 2018, but a large increase was seen in 2019. This abrupt spike may have been caused by the COVID-19 outbreak, which made researchers more concerned about respiratory conditions like bronchiectasis. In general, the number of published papers in this field has an obvious binomial increase trend. After binomial fitting, the equation is *y* = 1.7452*x*^2^ + 30.942*x* + 6.3184 and the R square equals 0.9998, which represents an excellent binomial fitting degree (Figure 2). According to the logical curve model proposed by Price, research on pediatric bronchiectasis seems at the initial part of the second stage of literature growth, that is, the period of rapid development. At this stage, professional theories develop rapidly, and the number of papers increases sharply, strictly obeying the law of exponential growth. More attention has been paid to pediatric bronchiectasis in clinical or basic research. From the growth curve, it can be predicted that the research in this field will continue to rise and is one of the current hotspots in pediatrics, while the theory and research methods need to be further studied.

### Analysis of countries/regions and institutions

**Figure 3:**
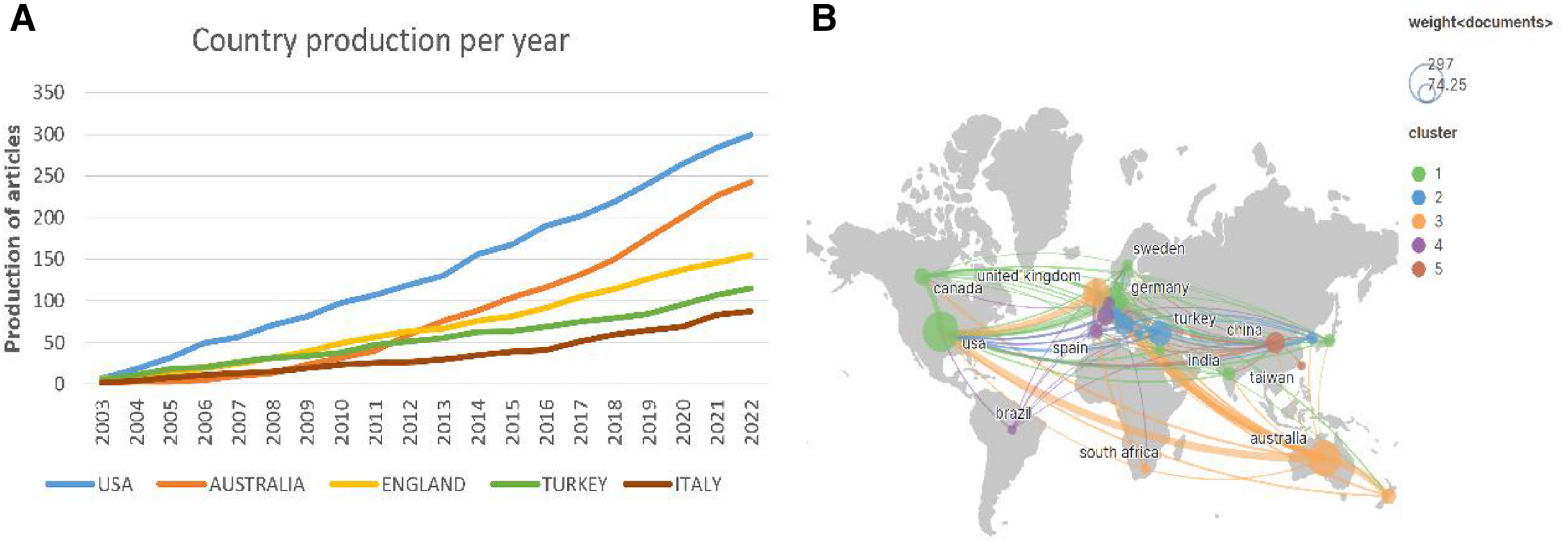
Analysis of countries/regions related to pediatric bronchiectasis between 2003 and 2022. (**A**) Trend chart of the number of publications in the top five countries. (**B**) Cooperation network map of countries. Node size represents the number of publications. The lines represent cooperation relationships, and the thickness of the line represents the connection strength.

In total, 80 countries/regions contributed to the 1,351 articles on pediatric bronchiectasis worldwide, among which the United States (*n* = 299, 22.13%) contributed the most, followed by Australia (*n* = 243, 17.99%) and England (*n* = 155, 11.47%). Among the top ten nations, four-fifths were developed countries. As shown in the line graph (Figure 3A), the number of publications in Australia increased rapidly mainly from 2011 to 2012, becoming the second most productive country after the United States, while those in other countries grew steadily over the years. The top five countries/regions with total citations were the United States (*n* = 9,800), Australia (*n* = 7,763), England (*n* = 6,145), Netherlands (*n* = 3,186), and Italy (*n* = 2,039). Both Iceland and Slovenia had only one article, but the numbers of citations were 627 and 223, respectively, with the average number of citations ranking the first (*n* = 627) and the second (*n* = 223). Although Chile was not in the top five list in terms of the publication number or total citation number, the average number of citations ranked third (*n* = 113.8). Additionally, according to the extensive cooperation between countries/regions illustrated in Figure 3B, the number of collaborators with the United States was 41, with a total link strength of 198, which had the most frequent cooperation with other countries, followed by England (links = 36, total link strength (TLS) = 231) and Australia (links = 31, TLS = 169).

**Figure 4:**
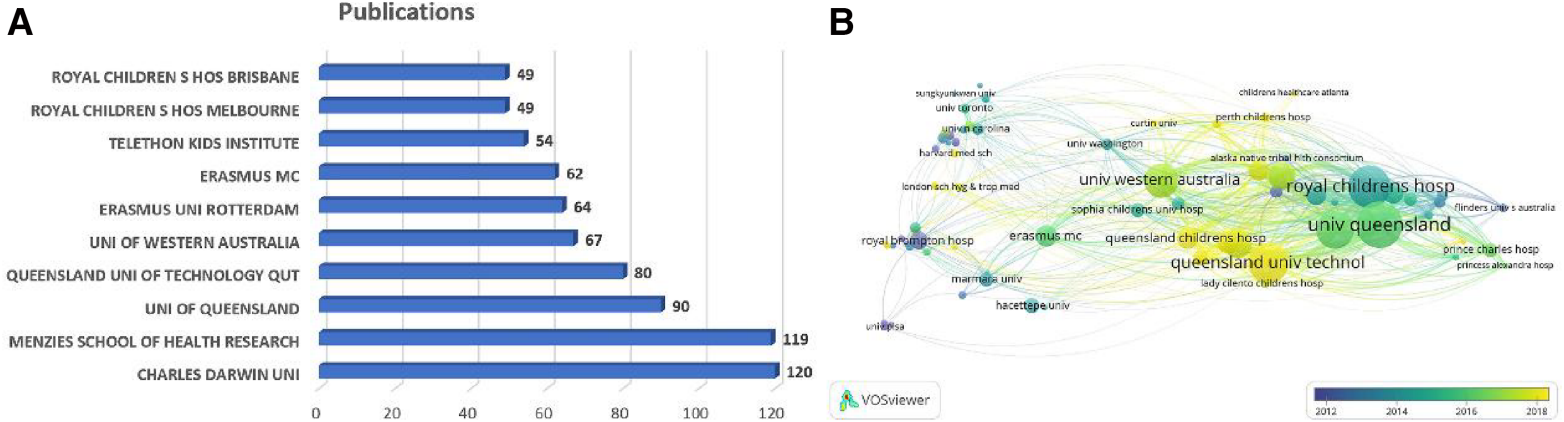
Analysis of institutions related to pediatric bronchiectasis between 2003 and 2022. (**A**) Bar chart of the number of publications in the top 10 institutions. (**B**) Cooperation network map of institutions. Node size represents the number of publications, link represents the cooperation relationship, and color represents the average cooperation year.

The included articles were contributed by 1,865 institutions. The top five institutions accounted for 476 articles (35.26%, Figure 4A), which were Charles Darwin University (*n* = 120, 8.89%), Menzies School of Health Research (*n* = 119, 8.81%), University of Queensland (*n* = 90, 6.67%), Queensland University of Technology (*n* = 80, 5.93%), and Erasmus University Rotterdam (*n* = 67, 4.96%). It is noteworthy that seven out of the top 10 institutions are located in Australia. Furthermore, we analyzed the institutional co-occurrence map through VOSviewer, in which the node size represents the number of citations and the link represents the co-cited relationship. The cooperation network map in Figure 4B shows the dense connections within institutions, among which the University of Queensland, Charles Darwin University, and Royal Children's Hospital collaborated most frequently with others, with centrality degrees of 52, 51, and 46, respectively. That is to say, these institutions were fully devoted to children bronchiectasis research, working as hinge nodes to connect others. However, connections between institutions in different countries are still weak, which enlightens us that institutions must prioritize improving interdisciplinary collaboration.

### Analysis of journals

There are 384 journals that published articles on pediatric bronchiectasis, and 67 of them have published more than 5 articles. Table 2 lists the top ten journals that had published 382 articles in total, accounting for 30.25% of all. Among the top ten journals with the most publications, *Pediatric Pulmonology* [*n* = 149, 11.80%, impact factor (IF) 2021 = 4.09] was the biggest contributor and had the total citations of 3,068, but its average citation count was only 20.59, while *New England Journal of Medicine* had the highest average cited frequency of 389.5. *Chest* (*n* = 45, 3.56%, IF 2021 = 10.262) and *European Respiratory Journal* (*n* = 33, 2.61%, IF 2021 = 33.795) ranked second and third, respectively. American and British journals accounted for four each of the top ten productive journals. Simultaneously, the IF of these 10 journals is between 3.569 and 33.795. There was one journal with IF < 4.000, six journals with IF between 4.000 and 10.000, and three journals with IF > 10.000. Additionally, 7 out of the top 10 journals were in the Q1 JCR region, and the IF of the *European Respiratory Journal* (*n* = 33, 2.61%, IF 2021 = 33.795) was the highest.

**Table 2:** Top 10 journals with publications related to bronchiectasis in children between 2003 and 2022.

Rank	Journal	Publications	Total times cited	Mean times cited	Impact factor (2021)	JCR
1	*Pediatric Pulmonology*	152	2,794	18.38	4.090	Q2
2	*Chest*	44	2,194	49.86	10.262	Q1
3	*European Respiratory Journal*	34	1,792	52.71	33.795	Q1
4	*Journal of Cystic Fibrosis*	29	444	15.31	5.527	Q1
5	*American Journal of Respiratory and Critical Care Medicine*	25	3,264	130.56	30.528	Q1
6	*Respiratory Medicine*	25	701	28.04	4.582	Q2
7	*Pediatric Respiratory Reviews*	23	355	15.43	5.526	Q2
8	*Frontiers in Pediatrics*	22	74	3.36	3.569	Q1
9	*Thorax*	21	1,849	88.05	9.102	Q1
10	*Respirology*	18	279	15.5	6.175	Q1

**Figure 5:**
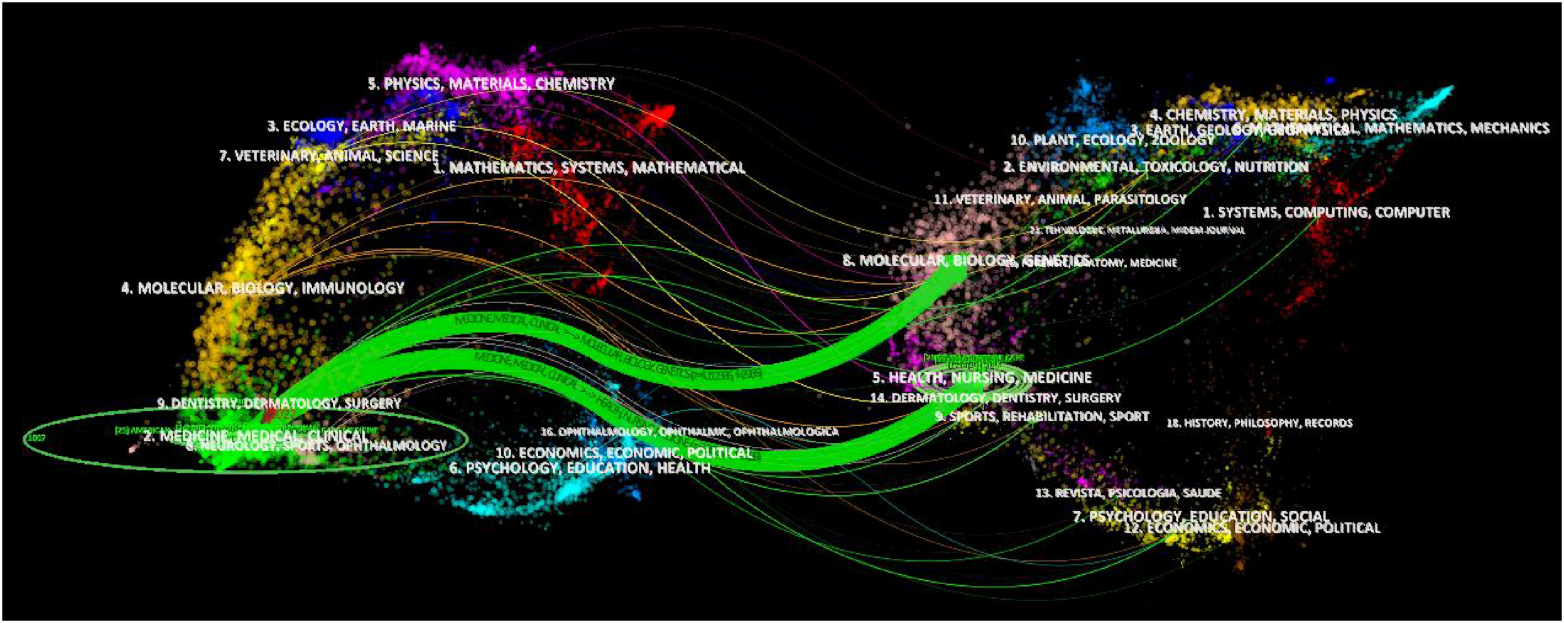
Dual-map overlay of journals related to pediatric bronchiectasis between 2003 and 2022. Citing journals are on the left, and cited journals are on the right. The horizontal axis of the ellipse represents the number of authors, and the vertical axis represents the number of publications. The lines depict the citation paths.

The dual-map overlay of journals indicates the topic distribution of the academic journals and can analyze the citation relationship between journals, with the citing journals on the left and the cited journals on the right, reflecting the discipline changes (36). The left can be seen as the application field, and the right can be seen as the research base. The center of the ellipse represents the topic of a specific journal. The horizontal axis of the ellipse represents the number of authors, and the vertical axis represents the number of publications. The lines depict the citation paths. As shown in Figure 5, there are two main reference paths indicating that studies published in molecular biology/genetics and health/nursing/medicine journals are frequently cited by studies in medicine/medical/clinical journals. Journal analysis can provide a direction for the authors to submit their articles in the future.

### Analysis of authors and cooperative relationships

**Figure 6:**
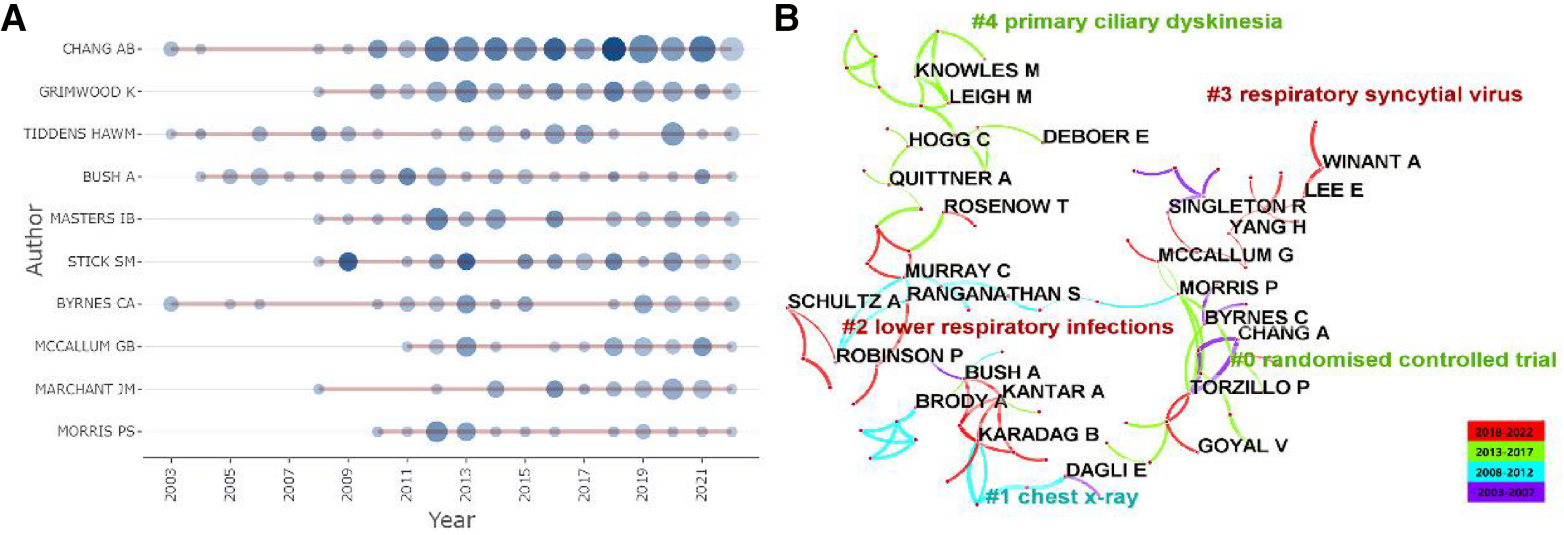
Analysis of authors related to pediatric bronchiectasis between 2003 and 2022. (**A**) Annual publication and citation count of top 10 authors. Node size represents the number of publications, and the shade of the color represents the number of citations. (**B**) Cooperation network map of authors, in which collaboration groups and the top five keyword clusters are shown. Link thickness represents the cooperation strength.

A total of 1,351 articles were published by 6,593 authors, and the average number of authors per paper was 5.11. As listed in Table 3, 397 articles were contributed by the top 10 most productive authors, accounting for 29.39% of all publications, and 7 out of them were from Australia. The top three most published authors were Chang AB (*n* = 115, 8.52%), Grimwood K (*n* = 49, 3.63%), and Tiddens HAWM (*n* = 37, 2.74%). In contrast, for the average citation count, the top three authors were Gangell CL (*n* = 230.83), Murray C (*n* = 123.50), and Sly PD (*n* = 93.39). It is worth mentioning that Chang AB [Menzies School of Health Research (MSHR)], the most prolific author in this field, has been exploring the bronchoscopic findings in children with chronic suppurative lung disease since 2002, which revealed bronchoscopic manifestations of bronchodilation. As displayed in Figure 6A, we could rapidly grasp the information that Chang AB has maintained a high level of issuance of about 10 papers per year since 2012. An average citation of 63.6 of nine papers published in 2018 was shown as the circle in the darkest color. Significantly, Chang AB, the most prolific author, also had the total citation number of 3,164, followed by Stick SM, with a total citation number of 2,230. As exhibited in Figure 6B, after keyword clustering of the generated cooperative network graph, there were close cooperations among authors in different clusters. The three authors with the highest centrality were Chang AB (67), Grimwood K (49), and McCallum GB (40), who had cooperated most frequently with other authors and invested much effort in the study of bronchiectasis in children with lower respiratory infections, especially by the respiratory syncytial virus, indicating that they may have formed a basic cooperation network. However, in the same collaboration network, most authors were from the same country, while authors from different countries in this field were not closely connected in general. To promote this subject, it is suggested that future coordination between academics from various nations might be enhanced.

**Table 3:** Top 10 authors and co-cited authors of bronchiectasis in children between 2003 and 2022.

Rank	Author	No. of publications	No. of citations	Mean times cited per article
1	Chang AB	115	3,176	27.15
2	Grimwood K	49	494	38
3	Tiddens HAWM	37	1,951	39.82
4	Bush A	32	1,126	48.96
5	Masters IB	32	516	27.16
6	Stick SM	31	2,230	73.94
7	Byrnes CA	29	1,109	35.77
8	McCallum GB	26	478	23.9
9	Marchant JM	25	743	23.97
10	Morris PS	21	737	35.1

### Analysis of keywords

**Figure 7:**
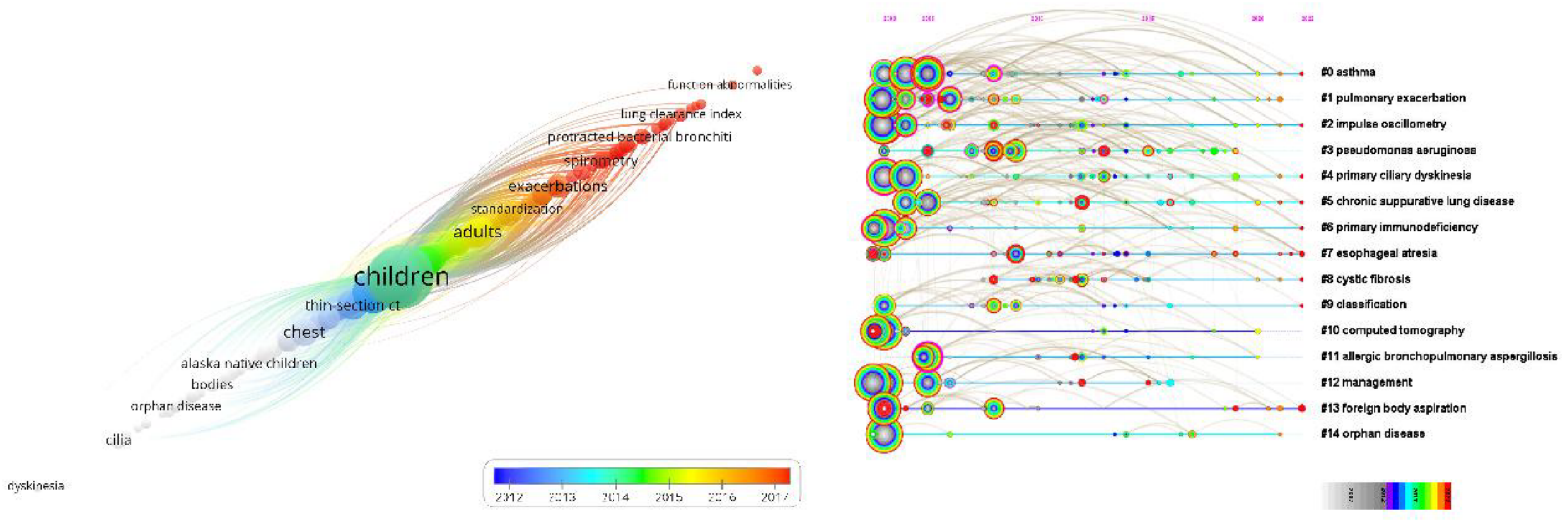
Analysis of keywords of bronchiectasis in children between 2003 and 2022. (**A**) Keyword evolution graph. Node size represents the frequency, and different color represents different years, as the label shows. (**B**) Timeline visualization of keywords. Nodes of the same cluster are arranged in the same row from left to right in chronological order. Nodes with purple outer rings are burst nodes.

**Figure 8:**
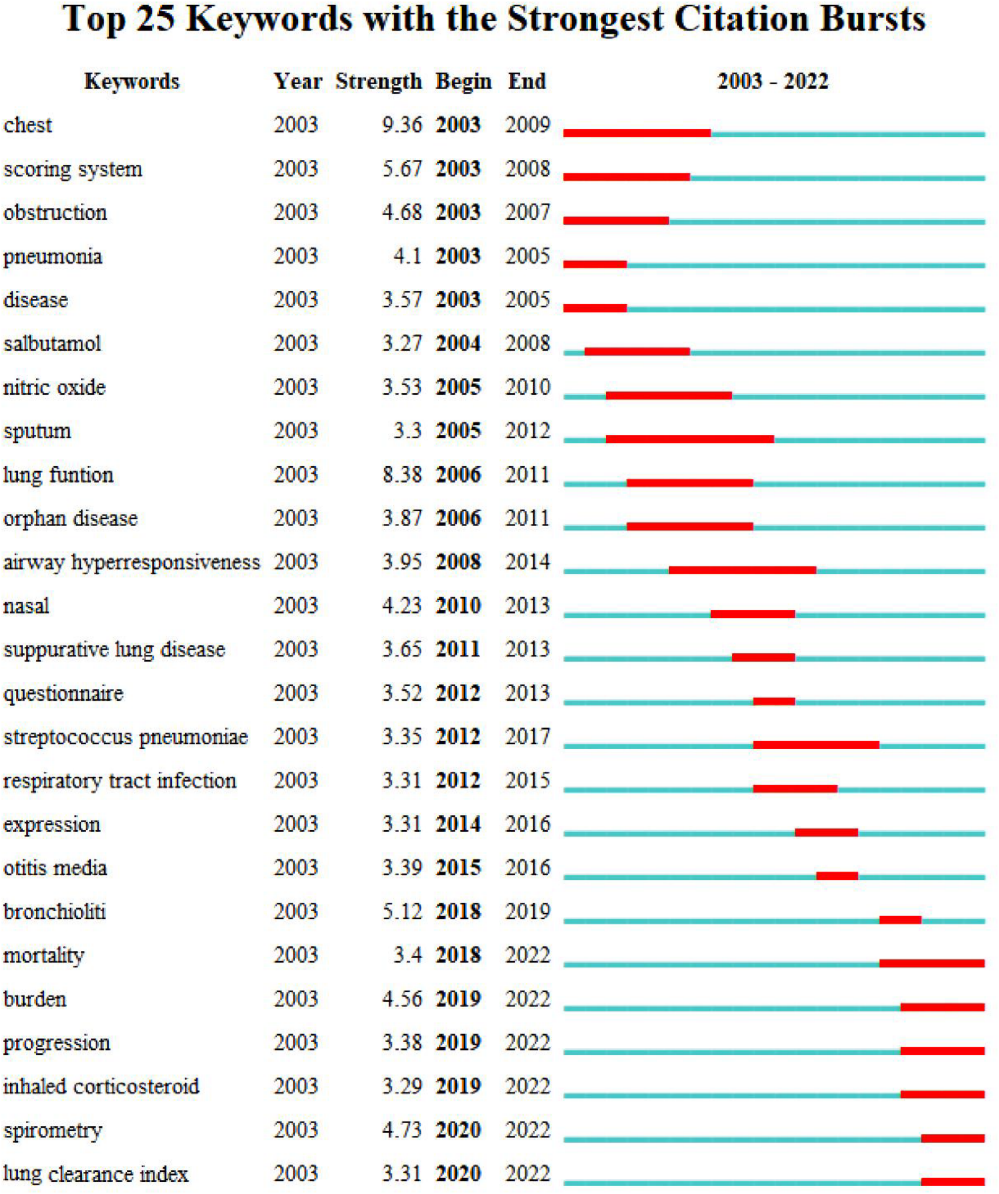
Top 25 keywords with the strongest citation bursts.

As the core of an article, keywords refine the content, providing readers with the primary topic quickly. As listed in Table 4, the 10 keywords that appeared most were “children” (*n* = 757), “cystic fibrosis” (*n* = 381), “bronchiectasis” (*n* = 283), “ct” (*n* = 269), “pulmonary-function” (*n* = 250), “disease” (*n* = 230), “pulmonary disease” (*n* = 217), “infection” (*n* = 186), “diagnosis” (*n* = 134), and “asthma” (*n* = 128). The top 10 keywords with the highest degree of centrality were “Airway hyperresponsiveness” (0.22), “Disorder” (0.22), “Airway” (0.20), “Bronchoalveolar lavage” (0.20), “Prevalence” (0.19), “Randomized controlled trial” (0.18), “Abnormality” (0.18), “Cough” (0.18), “Age” (0.17), and “Management” (0.16), which had the closest relationships with other keywords. The keyword evolution graph can effectively convey information on the development of hot themes over time, offering guidance for future research. In the keyword evolution graph, node size represents the frequency of occurrence and different colors represent different years, as shown by the label below the figure. As presented in Figure 7A, “lung clearance index,” “function abnormalities,” and “spirometry” were research hotspots emerging gradually. However, the research on dyskinesia and cilia has slightly slowed down in recent years. Clustering is a classification method of categorizing keywords by some common characteristics, simplifying the complex network to present a more understandable depiction for researchers (37). Keywords’ timeline visualization in Figure 7B was generated by CiteSpace 6.1.R2, in which the words with “# number” on the right represent the order/importance and the name of the keyword cluster. It presents the evolution of scientific knowledge and simplifies complex relationships by clustering analysis (38). The keywords of bronchiectasis in children were classified into 17 clusters, while the biggest 15 clusters were shown. The sequence number was ranked according to the cluster size, in the following order: “asthma”#0, “pulmonary exacerbation”#1, “impulse oscillometry”#2, “pseudomonas aeruginosa”#3, “primary ciliary dyskinesia”#4, “chronic suppurative lung disease”#5, “primary immunodeficiency”#6, “esophageal atresia”#7, “cystic fibrosis”#8, “classification”#9, “computed tomography”#10, “allergic bronchopulmonary aspergillosis”#11, “management”#12, “foreign body aspiration”#13, “orphan disease”#14, “disease”#15, and “quality of life”#16. Various colors were used to differentiate clusters clearly. Keyword timeline analysis mainly focused on describing the relationships between clusters and the historical span of keywords in each cluster, clarifying the research history of bronchiectasis in children. In addition, the nodes of the same cluster were arranged on the same horizontal line in chronological order. The time was placed at the top of the view (the closer to the right, the closer to the present time) (39). It is demonstrated in Figure 7B that the clustering of “asthma,” “pseudomonas aeruginosa,” “primary immunodeficiency,” “cystic fibrosis,” and “computed tomography” spanned a long period of time, which denotes that they have maintained the research cores in this field. In other words, they have occupied important positions in this field and have formed stable research directions. In addition, the research on “chronic suppurative lung disease” still remained popular. “Pulmonary exacerbation” and “impulse oscillometry” were hotspots merging in recent years. To further investigate the hotspots in this field, CiteSpace 6.1.R2 was employed to identify and analyze keywords with strong citation bursts in pediatric bronchiectasis, which may serve as a crucial indicator of future hotspots. Burst detection revealed keywords that increase abruptly over time, which can reflect the changes and developing trends of research direction in a certain field to some extent. As illustrated in Figure 8, the blue line represents the time interval and the red line represents the burst period of the keyword. The keywords with the strongest citation bursts included “chest” (2003–2009), “scoring system” (2003–2008), and “obstruction” (2003–2007), while the most recent keywords with the strongest citation bursts were “spirometry” (2020–2022) and “lung clearance index” (2020–2022), revealing the ongoing forefront of this field and may continue to develop over time.

**Table 4:** Top 10 keyword co-occurrence frequency and centrality ranking.

Rank	Keyword	Frequency	Rank	Keyword	Centrality
1	Children	757	1	Airway hyperresponsiveness	0.22
2	Cystic fibrosis	381	2	Disorder	0.22
3	Bronchiectasis	283	3	Airway	0.20
4	CT	269	4	Bronchoalveolar lavage	0.20
5	Pulmonary-function	250	5	Prevalence	0.19
6	Disease	230	6	Randomized controlled trial	0.18
7	Pulmonary disease	217	7	Abnormality	0.18
8	Infection	186	8	Cough	0.18
9	Diagnosis	134	9	Age	0.17
10	Asthma	128	10	Management	0.16

## Discussion

As far as we know, there have been no bibliometric publications in this field, so this may be the first article in the field of pediatric bronchiectasis to perform a bibliometric analysis of research hotspots systematically with intuitively visualized figures to discover the current research condition and provide potential indications for future research directions, serving as a comprehensive guide for clinicians and academics working in this field.

### General information

In general, the annual number of publications on pediatric bronchiectasis has an increasing trend. The growth rate has increased significantly in recent years, indicating that there is still much uncertainty worth further exploring.

An in-depth analysis of countries, institutions, journals, authors, and keywords was conducted by combining multiple visualization and analysis software. The United States, Australia, and England were the top three most productive countries. It is worth noting that Australia’s publications have grown rapidly in recent years, with a high total citation count and dense collaboration with other countries. Generally speaking, countries with higher publication counts also have higher citation numbers, but there are also some exceptions, indicating high-quality articles, which need to be paid more attention. For instance, there was only one article in this field published in Slovenia, but the citation number was 223. The article revealed that activated phosphoinositide 3-kinase delta syndrome (APDS), a combined immunodeficiency with multiple clinical manifestations, had high rates of mosaic attenuation (90%) and bronchiectasis (60%) in thoracic imaging, while selective PI3K*δ* inhibitors were likely to provide a novel treatment direction (40). In terms of international cooperation, England has the most frequent cooperation with other countries. Despite this, the top four most published institutions and the top three institutions with the highest degree of centrality were all located in Australia, implying that Australia may have established a robust research network in this area. However, as we can see in Figure 4B, the lines between institutions located in the same country are thicker and numerous, while the lines between institutions in different countries are relatively sparser. It can be shown that cooperations among institutions in different countries were still weak, which needs to be strengthened to promote long-term development in the future.

In addition, not only were seven out of the top 10 institutions located in Australia but also 7 out of the top 10 productive authors came from Australia. Hence, the strong scientific research capacity of Australia in this field has been further proven. Chang AB, with the most citations and the highest degree of centrality, is also the most productive author in this field. It is worth mentioning that Chang AB has been researching bronchiectasis in children since 2002 (41). The average citation of Chang AB peaked in 2018 during nearly 20 years. One study, which was cited the most, suggested that prompt identification and optimal management of children with bronchiectasis were particularly significant (24), while another, which was cited the second, indicated that identifying bronchiectasis through the emerging phenotyping and endotyping techniques may achieve better outcomes (20). What is more, Chang AB found that bronchiectasis was a serious and common reason for children's chronic coughs. Early diagnosis and management could reduce respiratory morbidity in the future (42). They are all influential articles in this field. It is clear that Chang AB devoted herself to researching pediatric bronchiectasis and achieved significant advances in this field. Gangell CL, who had the highest average citations, was dedicated to the early diagnosis of cystic fibrosis through newborn screening (NBS) (43) and bronchoalveolar lavage (BAL) (44). However, authors from the same country seemed to cooperate more, while the international collaboration of authors remained to improve to promote in-depth research for further development.

Among the top 10 journals with the most publications, *Pediatric Pulmonology* ranked first, reflecting its global influence on the field of pediatric bronchiectasis. The *New England Journal of Medicine* had the highest average cited frequency. Specifically, Sly et al. found that neutrophil elastase activity in BAL fluid in early life was relevant to bronchiectasis in children with cystic fibrosis, which was published in the *New England Journal of Medicine* (44). *European Respiratory Journal* has the highest IF among the top 10 productive journals, while the *American Journal of Respiratory and Critical Care Medicine* ranks second but has the greatest total link strength. As illustrated in the dual-map overlay of journals, studies published in molecular/biology/genetics and health/nursing/medicine journals were frequently cited in studies published in medicine/medical/clinical journals.

### Analysis of keyword-based research hotspots

As can be observed in Table 4, the five most frequent keywords were “children,” “cystic fibrosis,” “bronchiectasis,” “ct,” and “pulmonary-function.” According to the keyword timeline map and heat map, “impulse oscillometry,” “bronchoalveolar lavage,” and “spirometry” had been frequently researched in recent years. The most recent keywords with the strongest citation bursts were “spirometry” and “lung clearance index.”

As for the keyword “cystic fibrosis,” CF is a genetic disorder caused by a defective cystic fibrosis transmembrane conductance regulator (CFTR) gene, which can lead to chronic multisystem disease including severe bronchiectasis (45). Meanwhile, lung involvement is the major cause of morbidity and mortality. The radiological evidence of bronchiectasis was detected in 50%–70% of children with CF before they reach school age (46). Meanwhile, it can appear as early as three months of age and develop most rapidly over the first 2–3 years of life (44). Antibiotics and airway clearance remain mainstays; in addition, hyperosmolar agents and mucolytics are used as adjuvant therapies (19). However, the focus of CF is shifting from treating established bronchiectasis to preventing bronchiectasis and delaying the onset of lung disease (47). Notably, with the Food and Drug Administration approval of the first CFTR modulator ivacaftor in 2012, which has been proven to significantly improve the quality of life of patients, the treatment of CF turned into a new era of precision medicine (48). Moreover, a basic study showed that CFTR activity could be restored, indicating a promising therapeutic strategy (49). However, given the exorbitant prices, patients with no access to CFTR modulators urgently need a new effective alternative therapy, and the potential side effects and drug–drug interactions still require careful follow-ups in the coming years (50). Although there are many differences between CF and non-CF, like etiologies, clinical and microbiologic features, and antibiotic sensitivity, the management of non-CF bronchiectasis in children has long been based on CF and studies in adults (51, 52). However, non-CFTRs have recently been found to have dysfunction to some degree (53), indicating that CF treatment such as CFTR modulators may also be feasible for non-CF. However, the efficacy and feasibility should be further confirmed.

The biochemical examination of BAL is still widely recognized as the golden standard for detecting endobronchial infections relevant to lower respiratory inflammation, especially in CF. Nevertheless, this is not only because bronchoscopy with BAL does not provide as much useful information as other evaluation methods, but it is also very invasive; European Respiratory Society (ERS) guidelines recommend it as a selective rather than a necessary means (23, 54). Given that clinical manifestations of most children with structural pulmonary disease are not obvious and the current care mode fails to prevent respiratory sequelae, free neutrophil elastase activity in BAL fluid was closely related to bronchiectasis, so it can be the predictor in the early life of children with cystic fibrosis (44, 46, 55). Overall, it is less likely that bronchoalveolar lavage becomes the first choice for diagnosis, while it remains to be investigated whether BAL is sensitive enough to be recommended for early screening in special populations with high-risk factors.

The “pulmonary-function” and “ct” are both popular keywords regarding diagnosis and assessment. Although the Bronchiectasis Severity Index (BSI), FACED, and E-FACED scores have been developed to assess the bronchiectasis severity, accurate scoring requires a long period of follow-up (56–58). However, spirometry and impulse oscillometry can evaluate the bronchiectasis severity at the first visit (59), but the sensitivity remains uncertain. As a recognized diagnostic method and an estimation means for the severity of bronchiectasis, HRCT offers specific imaging indices, including bronchoarterial ratio, bronchial wall thickness, bronchial structures, the presence of mucus plugs or mosaic perfusion, and other imaging markers. High-resolution multidetector CT (MDCT) is suggested in the European guidelines as the standard technique for diagnosing bronchiectasis in children due to its higher sensitivity than conventional HRCT (60). On the contrary, the British guidelines suggest that a standard HRCT examination is sufficient to show bronchiectasis (61). Although radiation exposure could be reduced by modified low-dose CT, it cannot be eliminated. Therefore, studies suggested MRI as an appropriate alternative method (62–64). MRI is free of radiation and better at detecting early structural or functional changes in local lungs (65, 66), while the most significant drawbacks are the increased time and cost. Overall, it seems hard for MRI to replace CT in pediatric bronchiectasis after comprehensively evaluating its advantages and disadvantages; nonetheless, it is still worth investigating under what circumstances MRI can bring additional benefits.

“*Haemophilus influenzae*” and “*Pseudomonas aeruginosa*” are common major pathogens of pediatric bronchiectasis and also the independent predictors of acute exacerbations (67). Recent research found that nontypeable *H. influenzae* (NTHi) is the most common bacterial pathogen identified in BAL fluid from both adult (32%) and pediatric (47%) patients (68) rather than *P. aeruginosa*. Currently, published placebo-containing randomized controlled trials evaluating the efficacy of the antibiotics in exacerbations are sparse. Although guidelines retain inhaled antibiotics as an optional treatment for acute exacerbations, their efficacy and duration still require further investigation. In addition to inhaled antibiotics, amoxicillin clavulanate is considered a first-line empirical oral antibiotic in European guidelines for both adults and children (5, 23). Compared with azithromycin, amoxicillin clavulanate performs better in relieving symptoms (69, 70). However, azithromycin could reduce the exacerbation duration more than amoxicillin clavulanate, and long-term azithromycin notably reduces exacerbation rates (71). Because children have poorer adherence, there are research studies indicating that azithromycin has the potential of replacing amoxicillin clavulanate as the first-line antibiotic for its longer half-life and more convenient dosing schedule, which made “azithromycin” a hotspot in recent 20 years. However, in addition to the differences in symptom relief, more evaluation indicators such as time-to-next exacerbation, quality of life, and hospitalizations should be taken into consideration, and more high-quality RCTs are required, which may be a research direction for research in the next few years.

What is more, studies have shown that children with a history of childhood bronchiectasis are more likely to experience persistent respiratory symptoms, growth failure, and developmental delay in the future. They are also more likely to develop other respiratory diseases incorporating asthma, COPD, and cor pulmonale in adulthood (72–74). Although the adult outcome of pediatric bronchiectasis is a critical issue that cannot be ignored, there is too little case data to support sufficient research due to the low detection rate of childhood bronchiectasis in previous times. According to the results of keyword analysis, there are many unanswered questions about childhood bronchiectasis, and more research is needed to find answers to these questions.

## Strengths and limitations

To our knowledge, this is the first study to perform bibliometric analysis and visual display of pediatric bronchiectasis. We included the vast majority of articles published in this field in recent 20 years and analyzed the retrieved data systematically by bibliometric software to intuitively show the current research trend, which could offer significant indications and ideas for researchers concerned with this field. However, there were still certain inevitable limitations. First, because our retrieved publications were all from WoSCC, articles from other databases were not involved. Second, publications in other languages were missed since articles written in only English were selected in our study. Third, subtle analysis discrepancies may exist in different bibliometric software, which might result in bias in other bibliometric analyses.

## Conclusion

Through rigorous and thorough bibliometric analysis, this study reveals the current status of pediatric bronchiectasis, assisting researchers in grasping research hotspots and frontiers in this field and thus exploring potential novel directions for further in-depth research. The United States contributes most in publications, while Australia has developed rapidly and provided a pivotal influence in this field in recent years. Chang AB is the most prolific author with the highest total citation count.

Work on pediatric bronchiectasis has made considerable progress in the last two decades, but there are still many unresolved issues. The current research hotspots of pediatric bronchiectasis have been focused on the etiopathogenesis, early diagnosis, severity assessment, prediction, prevention and treatment of acute exacerbations, and, most importantly, therapeutic interventions and optimal long-term management to prevent, slow, or even reverse the progression of bronchiectasis in children, which may remain the research trends and forefront topics in the following years.

## Data Availability

The original contributions presented in the study are included in the article/Supplementary Material, further inquiries can be directed to the corresponding author.
